# A case report of vibration-induced hand comorbidities in a postwoman

**DOI:** 10.1186/1471-2474-12-47

**Published:** 2011-02-14

**Authors:** Stefano Mattioli, Francesca Graziosi, Roberta Bonfiglioli, Giuseppe Barbieri, Sandra Bernardelli, Luciano Acquafresca, Francesco S Violante, Andrea Farioli, Mats Hagberg

**Affiliations:** 1 Section of Occupational Medicine, Department of Internal Medicine, Geriatrics and Nephrology, University of Bologna, Bologna, Italy; 2 Department of Public Health, AUSL Bologna, Bologna, Italy; 3 Department of Public Health and Community Medicine, Occupational and Environmental Medicine, University of Gothenburg, Gothenburg, Sweden

## Abstract

**Background:**

Prolonged exposure to hand-transmitted vibration is associated with an increased occurrence of symptoms and signs of disorders in the vascular, neurological and osteoarticular systems of the upper limbs. However, the available epidemiological evidence is derived from studies on high vibration levels caused by vibratory tools, whereas little is known about possible upper limb disorders caused by chronic exposure to low vibration levels emitted by fixed sources.

**Case presentation:**

We present the case of a postwoman who delivered mail for 15 years using a low-powered motorcycle. The woman was in good health until 2002, when she was diagnosed with bilateral Raynaud's phenomenon. In March 2003 a bilateral carpal tunnel syndrome was electromyographically diagnosed; surgical treatment was ineffective. Further examinations in 2005 highlighted the presence of chronic tendonitis (right middle finger flexor).

**Risk assessment:**

From 1987, for 15 years, our patient rode her motorcycle for 4 h/day, carrying a load of 20-30 kg. For about a quarter of the time she drove over country roads. Using the information collected about the tasks carried out every day by the postwoman and some measurements performed on both handles of the motorcycle, as well as on both iron parts of the handlebars, we reconstructed the woman's previous exposure to hand-arm vibration. 8-hour energy-equivalent frequency weighted acceleration was about 2.4 m/s^2^. The lifetime dose was 1.5 × 10^9^(m^2^/s^4^)hd.

**Conclusions:**

The particular set of comorbidities presented by our patient suggests a common pathophysiological basis for all the diseases. Considering the level of exposure to vibrations and the lack of specific knowledge on the effects of vibration in women, we hypothesize an association between the work exposure and the onset of the diseases.

## Background

Hand-Arm Vibration (HAV) is defined as the transfer of vibration from a tool to a worker's hand and arm. The amount of HAV is characterized by the acceleration level of the tool when grasped by the worker. The vibration is typically measured on the handle of the tool to determine the acceleration levels transferred to the worker[1].

Prolonged exposure to hand-transmitted vibration is associated with an increased occurrence of symptoms and signs of disorders in the vascular, neurological and osteoarticular systems of the upper limbs[2]. The combination of these disorders is called Hand-arm Vibration Syndrome (HAVs).

Several epidemiologic studies have been conducted to establish exposure-response relationships between hand-transmitted vibration and disorders of the upper extremities. The relationship between white finger and vibration exposure has been extensively investigated, whereas there is a shortage of exposure and epidemiologic data for vibration-induced neurological and musculoskeletal disorders[3].

Many studies conducted among manual workers (quarry drillers, stone carvers, platers, truck assemblers, forestry workers [4-10]) have been published in the scientific literature. However, little is known on signs and symptoms related to segmental vibration exposure in motorcyclists[11].

For example, it is unclear from current evidence whether the increased risk of carpal tunnel syndrome (CTS) extends to fixed sources of hand-transmitted vibration as well as hand-held vibratory tools[12].

We encountered a case of multiple morbidities (bilateral CTS, Raynaud's phenomenon, and tendin involvement) in a woman previously exposed to HAV due to motorcycle riding.

## Case Presentation

We present the case of a woman employed as a postal worker in Italy, suffering from multiple comorbidities of the hand. The woman was first examined in this operative unit in 2004 at the age of 46. Her general state of health was good; her medical history revealed only a cholecystectomy, due to cholelithiasis, carried out in 2000. In 2002, due to the worsening of pain and paresthesia of the hands, the woman underwent rheumatological and vascular angiological examinations. At nailfold capillaroscopy, the architecture of vessels was regular and the loops were normally spaced and with a hairpin appearance; these findings, together with clinical examination and the self-reported history of multiple episodes of finger-blanching followed by intense redness led to a diagnosis of bilateral primary Raynaud's phenomenon. In March 2003, a bilateral carpal tunnel syndrome was diagnosed by electrophysiologic testing (moderate-severe on the right, moderate on the left). Neurolysis of the right median nerve did not prove effective as widespread dysesthesia across the hand, weakness and reduced dexterity were still present after the procedure, and pain at the first digit persisted. In 2005, further examinations highlighted the presence of chronic tendonitis of the right middle finger flexor (treated with tenolysis). In the same year, direct radiological examination identified initial occurrences of osteoarthritis in the distal interphalangeal joints of both thumbs; subsequent examinations carried out during 2007 found a worsening of the radiological status with a reduction of joint lines. At the last contact (2008), all the signs and symptoms described were still present and the patient complained of a progressive worsening of the pain.

On analyzing the risk factors possibly associated with the detected disorders, it emerged that the woman had a negative family history for the disorders under investigation: she was not affected by systemic diseases (scleroderma was explicitly ruled out by a rheumatologist after checking for blood levels of antitopoisomerase and antinuclear antibodies), she had normal thyroid functionality, she was not affected by diabetes or hyperlipidemia, she did not take medication regularly, she did not report any previous trauma of the upper limbs or the neck, she had never experienced frostbite injuries, she did not report any symptoms apart from those affecting the upper limbs, her weight was normal (body mass index between 21.4 and 23.6 kg/m^2 ^during the period under examination), she did not drink alcohol and only became a regular smoker in 2000 (10-15 cigarettes/day), and she was not exposed to any non-occupational activities which could correlate to possible pathological factors (hobbies, other working activities, sport, motorcycle riding outside work). On the basis of these findings, we decided to investigate occupational exposure, searching for possible risk factors.

## Risk assessment

**Figure 1:**
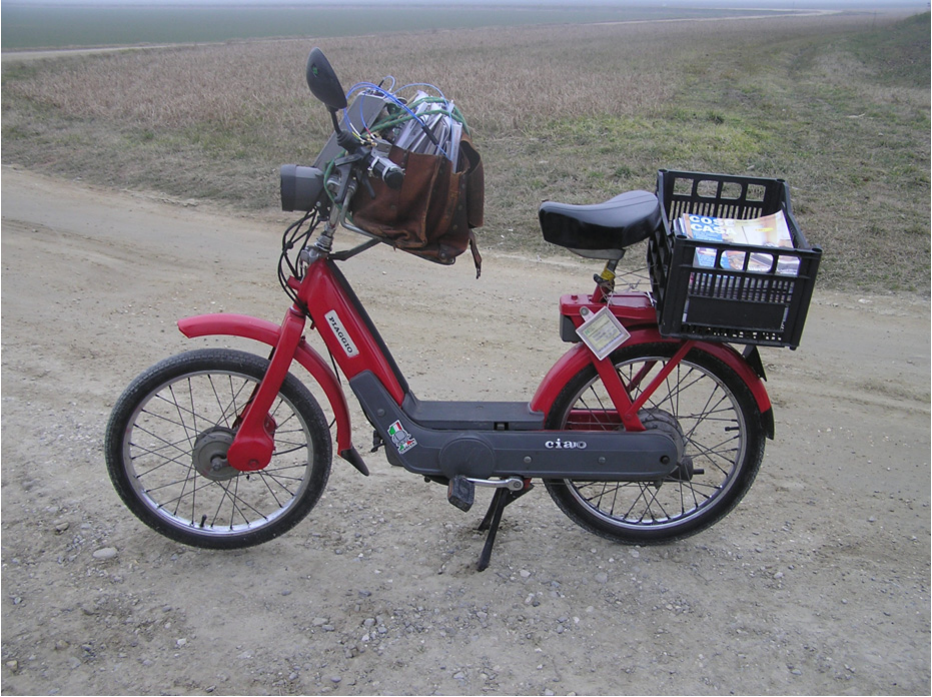
The motorcycle used by our patient to deliver mail (this photo was taken during our tests)

The woman had been working in a post office 6 h/day 6 days a week since 1982. From the start of her employment to 1987 she worked as a sorter; her activities consisted of sorting mail into pigeon-holes and in manual handling of mail bins. In this period she was exposed to biomechanical risk factors such as repetitive motions of the hands and fingers and pinch grip. From 1987 to 2002 she delivered mail using a motorcycle (Figure 1) and from 2002 to 2004 using a car. Due to her illness, she was reassigned to clerical duties in 2004. For 15 years the woman had driven a two-stroke 50 cc motorcycle to deliver mail and was exposed to hand-arm vibration for 4 h/day. According to the information collected, before delivering the mail, she had to prepare the mailbags by herself every day; this task consisted of checking that the mail was properly sorted and of filling her mailbags with 20-30 kg of mail. After these preliminary tasks, she carried out her main job-activity, consisting of delivering mail by riding her motorcycle for about 3 h/day on asphalted roads and for about 1 h/day on country roads, covering a distance of about 50 km/day. In the remaining work time, she did paperwork, thus avoiding exposure to biomechanical risk factors.

**Figure 2:**
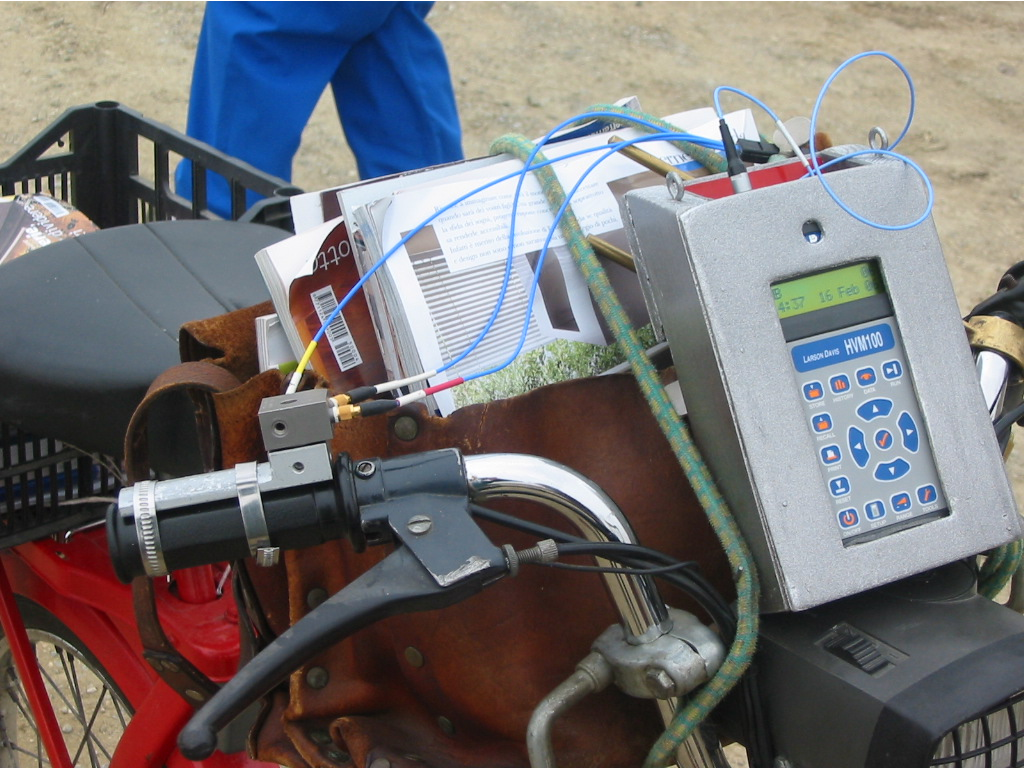
The accelerometer fastened to the handles (This photo was taken during our tests)

In order to evaluate whether and to what extent her work activity may have contributed to her condition, we performed some tests (16^th ^February, 2006) to measure the hand-arm vibration magnitude while the subject was riding the motorcycle. We used a Human Vibration Meter, model HVM 100 (Larson Davis Inc., USA) equipped with a tri-axial accelerometer (Bruel and Kjaer 4321), to measure the hand-arm vibrations in the orthogonal directions X, Y, and Z according to the International Standard ISO 5349 [13] (Figure 2).

The measurements were performed on both handles of the motorcycle as well as on both the iron parts of the handlebars. For vibration measurements on the handles, the accelerometer was firmly fastened to the handles as close as possible to the subject's hands, but not so close as to interfere with her activity. Tests were performed on two different road surfaces, loading the motorcycle with 2 cases (total weight 25 kg) mounted on the back and between the handlebars of the motorcycle. The results obtained are shown in Table 1.

**Table 1:** HAV measured on two different road surfaces

Handle		Iron handlebar		Vibration level (m/s^2^)	Type of Road	Measurement Time (s)
			
Right	Left	Right	Left			
		X		4.03	Country	191

		X		1.86	Asphalted	70

X				5.02	Country	185

X				2.65	Asphalted	63

	X			5.78	Country	160

	X			2.23	Asphalted	60

			X	4.47	Country	169

			X	1.85	Asphalted	61

The highest values on the handles corresponded to the country road. On the basis of the daily exposure period, including 1 h on a country road and 3 h on an asphalted road, the values for energy-equivalent frequency-weighted acceleration A(8) were 2.46 m/s^2 ^and 2.40 m/s^2 ^for the left and the right handle, respectively. These values are comparable with the exposure action level (A(8) = 2.5 m/s^2^) but lower than the daily exposure limit value (A(8) = 5.0 m/s^2^), as defined by European Directive 2002/44/CE, implemented by the Italian legislative decree 81/2008. The lifetime dose was 1.5 × 10^9 ^(m^2^/s^4^)hd, as calculated using the method suggested by Griffin[14]:

Lifetime dose = (∑(a_h_,_w_^2^t_h_)^1/2^t_d_t_y_)^2 ^((m^2^/s^4^)hd), where: a_h,w _is the frequency weighted acceleration, t_h _is the individually estimated daily exposure, t_d _the number of working days in a year and t_y _is the number of years of motorcycle use.

Ergonomic stress factors, as well as hand-arm vibration, are present when riding a motorcycle, like awkward postures of the wrist in combination with hand force. Flexions/extensions of the right wrist are associated with twisting the throttle handle to accelerate the motorcycle, while low grip force is required to hold the handlebars and finger force to press the brake levers. Moreover, during cold weather months the woman was also exposed to low temperatures.

## Discussion

The available literature does not report any similar cases of upper limb disorders associated with low-powered motorcycle riding. The patient was affected by neurologic, vascular and bilateral musculoskeletal disorders without systemic diseases, family history or non-occupational exposure to possible factors connected with the disorders described (hobbies, other working activities, sports). A detailed analysis of the subject's working activity allowed us to identify a risk factor correlated with all the disorders: for 15 years and for 70% of her working time (4 hours a day), the postal worker was exposed to vibrations transmitted to the hand-arm system by the use of a moped. Some studies have hypothesized a link between the use of motorcycles and disorders that involve hand strain. Nonetheless, these studies focus either on the levels of increased acceleration (the use of higher capacity motorcycles, travelling on rougher terrain) [15-17] or on histories of minor disorders [18,19]. The values obtained from the measurement of vibration exposure were comparable with the action threshold limits defined by European directive 2002/44/CE and by the Italian legislation (legislative decree 81/2008).

Some studies have shown an increased occurrence of signs and symptoms of entrapment neuropathies, mainly carpal tunnel syndrome, in occupations involving the use of vibrating tools [9,20]; of note, exposure to vibration is usually combined with other risk factors such as repetitive activity, awkward postures, heavy work. In the literature, the possible relationship between the use of motorcycles and the risk of developing CTS-like symptoms has been explored very little; two studies [10,15] indicate a correlation between the use of motorcycles and CTS-like symptoms. Sabeti-Aschraf et al.[16] hypothesize that motorcycling is likely to affect the median nerve. The acceleration values measured in this case-report are comparable with those found in other studies, measured on various types of equipment. For example, in Sutinen's article[6] the use of an old type chainsaw (1980's) for 3.5 h/day involved exposure to a level of vibration of 2.2 m/s^2^, while modern day chainsaws show a value of 1.8-2.2 m/s ^2 ^for prolonged exposure (4.5-5 h/day)[10].

Our patient was affected by bilateral Raynaud's phenomenon. We evaluated the risk of vibration-induced white finger using the formula reported in ISO standard 5349-1 [13]:

Dy/year = 31.8*(A(8)/m/s^2^)^-1.06^, where: Dy represents the duration of exposure.

A daily exposure of 2.4 m/s^2 ^results in a prevalence of disorders greater than 10%, after 12.5 years.

The comparison of the lifetime dose (values for our postal worker = 1.5*10^9^) with the results of a study conducted on forestry workers [7], underlines the surprisingly high value for our patient (comparable to the values of the maximum exposed group of forestry workers in the study by Bovenzi et al. [7]) and would seem to endorse the role of HAV in the development of the disorders described. We should, obviously, consider the substantial differences in terms of exposure (the postwoman was exposed to lower intensity levels of vibration for a longer time). Furthermore, all the previously cited studies were conducted on males doing heavy-duty work, as were the majority of vibration injuries investigations[21,22]. However, there are indications that vibration-induced injuries could be more common in women [23-25]. Compared with men, women seem to develop injuries after a shorter period of vibration exposure. Bylund showed that women had a high prevalence of vascular, neurological, muscular and skeletal symptoms, even though they had undergone low accumulated vibration exposure [21]. On this subject, the European Agency for Safety and Health at Work (2003) has recognised that work-related risks in women are generally underestimated and neglected both in research and prevention activities [26]. A common etiology can be suggested for the presence of bilateral CTS and Raynaud's phenomenon. Occupational exposure to vibrations seems, in this case, to be the most plausible explanation, even considering the absence of possible sources of non-occupational exposure. The current action limit may not be sufficiently protective; Hagberg[27] states that 2.5 m/s^2 ^"is not too low" and proposes a value of 1 m/s^2^. Our case-report seems to support Hagberg's statement and suggests the importance of studying gender differences in terms of dose-response to vibration exposure.

Ergonomic stress factors, as well as hand-arm vibration, are likely to have played a part in the development of various symptoms in the hand-arm system of the postwoman. In our subject, ergonomic problems arising while riding a motorcycle might be summarized as wrist posture (some degree of flexion/extension) and manual force, probably causing strain on the rider's hands and arms. Burstrom[28] reported that higher vibration levels as well as firmer hand grips could cause higher absorption in the human hand-arm system. Antivibration gloves represent the most common means of hand-arm vibration control for the receiver; however their effectiveness in abating hand-arm vibration from this kind of source (motorcycle riding) needs to be evaluated.

In addition to this, we should also underline that our patient worked as a sorter for 5 years at the beginning of her working life. Sorting can involve repetitive motions of the hands and fingers and low levels of pinch grip; a quite high (45.1%) one-month period prevalence of finger stiffness among female post office pensioners has been reported[29].

It is also important to consider the chronological onset of the symptoms. While finger blanching and pain at the fingers appeared when the postwoman was still exposed to vibration, the onset of tendon involvement was after exposure stopped.

Furthermore, the progression of the symptoms reported by our patient in 2008 is not typical for work-related musculoskeletal disorders.

However, symptoms of work-related diseases can be present many years after exposure cessation[29,30]. It is also possible to hypothesize the temporal overlapping of different risk factors: once the occupational factors of CTS and Raynaud's phenomenon were removed, age- and gender-related determinants could have played a role in the maintenance/worsening of the symptoms.

Furthermore, we should remember that female gender is a risk factor both for Raynaud's phenomenon and carpal tunnel syndrome; therefore all the exposures and disorders experienced by our patient could have occurred together by coincidence.

As regards osteoarthritic changes of the first digits, these are not uncommon in the general population[31]; therefore they may not be related to the other disorders reported here. Furthermore, osteoarthrosis of the fingers is not a typical disease for people exposed to HAV.

## Conclusions

This case report presents the hypothesis that the effect of HAV may result not in a single disorder, but in multiple disorders. Although occupational epidemiological studies do not usually address comorbidity, it is not uncommon for exposure to produce multiple effects (e.g. an association between HAV and tendonitis has been reported)[32]. Comorbidity due to occupational exposure therefore needs further epidemiological investigation.

## Consent

Written informed consent was obtained from the patient for publication of this case report and any accompanying images. A copy of the written consent is available for the review by the Editor-in-Chief of this journal.

## Competing interests

The authors declare that they have no competing interests.

## Authors' contributions

SM formulated the aetiological hypothesis, collected the clinical data and drafted the manuscript. FG perform exposure assessment, collected the exposure data and drafted the manuscript. RB collected the clinical data. GB perform exposure assessment and collected the exposure data. SB collected the exposure data. LA collected the exposure data. FSV revised the manuscript. AF drafted the manuscript and collected clinical data. MH revised the manuscript. All authors read and approved the final manuscript.

## Pre-publication history

The pre-publication history for this paper can be accessed here:

http://www.biomedcentral.com/1471-2474/12/47/prepub
